# Investigation of the potential of pomegranate peel as a treatment option for heavy metal contaminated wastewater: Experimental and modeling approaches

**DOI:** 10.1016/j.heliyon.2024.e34619

**Published:** 2024-07-14

**Authors:** Javad Zareei, Nizomiddin Juraev, Sabir Tagelsir Hassan Widatalla, M. Kerwad, Dmitry Olegovich Bokov, Khalid A. Alkhuzai, Carlos Rodriguez-Benites, Merwa Alhadrawi, Salah Hassan Zain Al-Abdeen

**Affiliations:** a Department of Biosystem Engineering, Ferdowsi University of Mashhad, Iran; b Faculty of Chemical Engineering, New Uzbekistan University, Tashkent, Uzbekistan; c Scientific and Innovation Department, Tashkent State Pedagogical University, Uzbekistan; d Department of Mathematics, Faculty of Science, University of Tabuk, Saudi Arabia; e General Department, Faculty of Information Technology, Misurata University, Misurata, Libya; f Institute of Pharmacy Named After A.P. Nelyubin, Sechenov First Moscow State Medical University, Russia; g Laboratory of Food Chemistry, Federal Research Center of Nutrition, Biotechnology and Food Safety, Russia; h Department of Civil Engineering, Al-Baha University, Al-Baha, Saudi Arabia; i Direccion de investigacion, Centro de investigacion de la Creatividad, Univesidad de Ciencias y artes de America Latina Lima, 15026, Peru; j Department of Refrigeration and Air Conditioning Techniques, College of Technical Engineering, The Islamic University, Najaf, Iraq; k Department of Refrigeration and Air Conditioning Techniques, College of Technical Engineering, The Islamic University of Al Diwaniyah, Al Diwaniyah, Iraq; l Department of Refrigeration and Air Conditioning Techniques, College of Technical Engineering, The Islamic University of Babylon, Babylon, Iraq; m Department of Medical Laboratories Technology, AL-Nisour University College, Baghdad, Iraq

**Keywords:** Adsorbent, Pomegranate peel, Cadmium, Mercury, Equilibrium, Thermodynamics

## Abstract

Heavy metals can cause serious environmental and human health problems, and their removal from wastewater is critical to protect our planet and communities. This study investigated the ability of crushed pomegranate peel to remove mercury and cadmium ions from contaminated water as a function of different experimental parameters. The experimental results showed that the pH of the solution influenced the adsorptive removal of heavy metals, with the best performance observed at pH 4.8. Optimization studies and process balance modeling were performed to optimize the process for commercial use. The performance of pomegranate peel was compared with that of other materials, and the highest adsorption capacities for both cadmium (Ca (II)) and mercury (Hg (II)) ions were observed to be 89.59 and 42.125 mg/g, respectively. The results were interpreted using the Langmuir model, which provided the best fit to describe the behavior of the process.

## 1 Introduction

Human industrial activities are the main cause of the accumulation of heavy metals in the environment [1]. They affect human well-being and can have serious and lasting effects on health and the ecosystem. Therefore, their impact should not be overlooked and we should all take steps to minimize their damage. The inability of heavy metals to biodegrade makes them a persistent threat to ecosystems and human life [2]. It is therefore imperative to find effective ways to treat heavy metal-contaminated water and prevent them from entering the human food chain [3,4].

Research into the potential of pomegranate peel (PGP) to remove heavy metals from contaminated wastewater is gaining popularity. It's a bio-sorbent that has been studied for its ability to capture a range of contaminants, including metals, dyes, and organic pollutants, its physical and chemical properties are known, and its absorption capacity has been evaluated under various experimental conditions [[5], [6], [7]]. Overall, the potential of pomegranate peel in wastewater remediation has gained attention in recent years and is expected to be an effective and environmentally friendly alternative [8,9]. The adsorption properties of PGP were compared with those of other agricultural waste biomass, highlighting its potential as a low-cost and effective substrate for wastewater treatment [[10], [11], [12], [13], [14]]. Experimental data on adsorption kinetics and isothermal models have been analyzed to understand the sorption behavior of PGP [15]. The potential of PGP for practical applications in wastewater treatment, including the removal of heavy metals, has been demonstrated [[16], [17], [18]]. Therefore, PGP shows promise as a renewable and cost-effective option for treating heavy metal-contaminated wastewater.

The removal of heavy metals from wastewater is a challenging task due to their persistence and poor degradation rates [19,20]. Traditional methods such as chemical precipitation and ion exchange are often expensive and lack selectivity [[21], [22], [23], [24]]. In addition, sedimentation methods lead to the production of toxic sludge, causing additional environmental problems [[25], [26], [27]]. Therefore, the search for alternative, cost-effective and environmentally friendly removal processes continues [28]. In this study, we investigated the potential of pomegranate peel as a novel substrate for the removal of mercury, cadmium and other heavy metals from wastewater [29,30]. This approach aims to reduce the environmental impact while providing an affordable and efficient solution for industrial operators.

With increasing attention being paid to biological absorption techniques, plants and plant parts have emerged as a cost-effective and environmentally friendly way to remove heavy metals from water and wastewater [31,32]. Pomegranate peel has shown particular promise as a high-capacity adsorbent, mainly due to its negative surface charge at high pH, which promotes the absorption of positive ions, including mercury and cadmium [33]. Unfortunately, the optimal operating conditions for these processes remain unknown and further research is required [[34], [35], [36]]. The proposed solution is to study the behavior of the adsorbent under different conditions and use the resulting equations to design industrial purification reactors [37]. In this way, we will be able to fully exploit the potential of pomegranate peel as a reliable adsorbent that can remove mercury and cadmium from polluted water or wastewater, leading to cleaner water and the possibility of reusing the separated metals [38].

A non-continuous method of removing heavy metals such as Hg (II) and Cd (II) from contaminated water involves mixing the adsorbent with the effluent in a turbulent pond. In this study, we developed a quasi-industrial scale reactor tailored for the purification of water loaded with these ions. Through meticulous equilibrium, kinetic, and thermodynamic calculations using this reactor, we have gained comprehensive insight into the absorption process. This effort allows us to determine the optimal conditions necessary for the efficient removal of each metal, a critical step toward industrializing the proposed process. In addition, our project has focused on assessing the technical and economic viability of using this approach for the removal of mercury and cadmium from industrial wastewater, as well as the potential for precious metal recovery.

## 2 Materials and methodology

### 2.1 Preparation of solution

Storage solutions were prepared by mixing mercury nitrate and cadmium nitrate (Merck, Germany) in 0.05 M nitric acid to a concentration of 1000 mg/L. The solution volume used for all experiments was 250 ml in 100 ml Erlenmeyer flasks, with 500 mg of crushed pomegranate peel added to absorb the metal ions. Before each experiment, the dishes were washed well by using a corrosive substance such as acid and then purified water solution. First, the pomegranate peel was mixed with 10 ml of distilled water for 15 min, then strained and mixed with 15 ml of 0.02 M acid for 15 min. To completely remove all traces of acid, the mixture was strained and then washed twice with 15 ml of distilled water. The metal ion solution and the adsorbent were shaken on a reciprocating shaker at an appropriate speed (RPM). The shaken solution was then passed through a filter paper, and the residual metal content was measured using a Flame Atomic Absorption Spectrometer (FAAS) Model 6800 manufactured by Shimadzu Factory in Japan. The temperature of the solutions was constantly controlled, and the pH of the solution was adjusted using soda or hydrochloric acid solutions. The pH of the solution was checked at each step using a pH meter, model PHS-3BW, manufactured by Bell Italy. The pH of the solutions was also checked at each step.

### 2.2 Preparation of chopped pomegranate peel

**Fig. 1:**
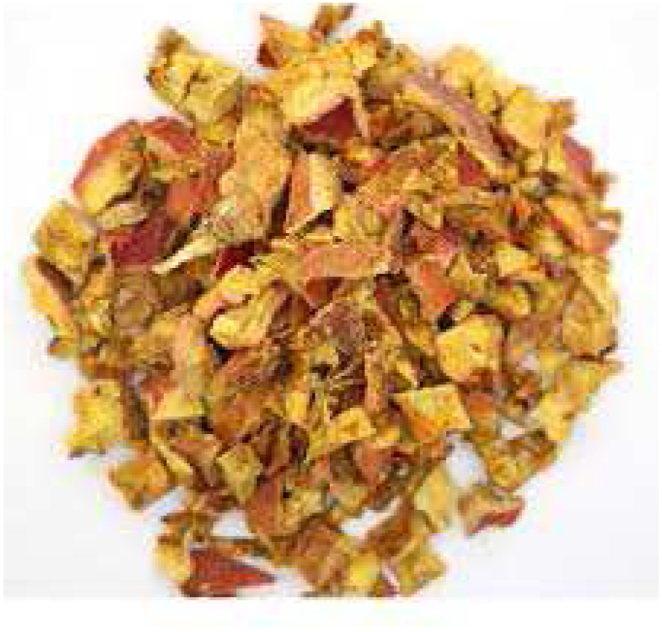
Dried pomegranate peel.

**Fig. 2:**
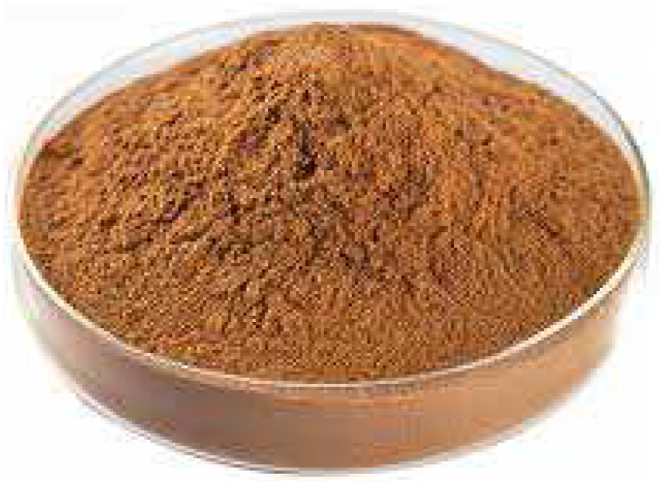
%100 Natural pomegranate peel powder.

Ionic storage solutions containing mercury and cadmium (1000 mg/Liter) were prepared using mercury nitrate and cadmium nitrate, both manufactured by Merck (Germany), in 0.05 M nitric acid. In all experiments, the volume of the solution used in 250 ml Erlenmeyer flask was considered as 100 ml, and 500 mg of crushed pomegranate peel was added for absorption tests. At each step, the dishes were washed well with 0.02 Molar acid and then with deionized water. In all experiments, the pomegranate peel was first mixed with 10 ml of distilled water for 15 min, strained, and then mixed with 15 ml of 0.02 Molar acid for 15 min. The mixture was strained and washed with 15 ml distilled water to remove the acid. In the next step, 100 ml of metal ion solution and adsorbent were mixed using a reciprocating shaker at a suitable speed (rpm). All the solutions were passed through a Whatman No. 40 filter paper, and the residual metal content was evaluated by a flame atomic absorption spectrometer model 6800 (manufactured by Shimadzu factory in Japan) at each stage of the investigation. The temperature of the solutions was closely monitored and the pH level was adjusted, if necessary, with either soda or hydrochloric acid solution. The pH level was measured during each step using a Bell Italy model PHS-3BW pH meter. Fig. 1 shows the dried pomegranate rind and Fig. 2 shows the all-natural pomegranate rind powder.

The quantitative analysis of some of the phytochemical compounds found in the pomegranate peel is presented in Table 1. The type of solvent used and the extraction process determine the amount of phenolic compounds, their ability to destroy free radicals, antibacterial activity and other biological functions of the pomegranate peel.

**Table 1:** Quantitative analysis of the phytochemicals present in the peel of the pomegranate.

Compound	Conc(mg/100 g)
Total phenolic content (gallic acid equivalent)	4842–6072
Total flavonoid content (quercetin equivalent)	518–852
Quercetin	5
Ferulic	5.2–6.13
Gallic acid	123.40–126.80
p-Coumaric acid	13–16.44
Ellagic acid	43.25–51
Catechin	840–872

### 2.3 Adsorption experiments

A batch system was used for the adsorptive experiments to determine the effect of pH on metal uptake, optimal incubation time, optimal isotherm models, metal sorption capacities, and thermodynamic calculations. The experiment was set up in 250 ml Erlenmeyer flasks with 500 mg of chopped pomegranate peel and shaken at an appropriate speed. After each test, the amount of mercury or cadmium remaining in the solution was determined using a flame atomic absorption spectrometer (FAAS) and used to calculate the degree of absorption in each experiment. The cadmium elimination percentage (Eq. (1)) was also determined and the amount of metal adsorbed were calculated (Eq. (2) and (3)) by subtracting the final concentration from the initial concentration and using the following formulas:

(1) $$R\left(\%\right)=\frac{C_{0}-C_{e}}{C_{o}}\times 100$$

(2) $$q_{e}=\frac{\left(C_{0}-C_{e}\right)V}{m}\;\text{The}\;\text{adsorption}\;\text{capability}\;\text{of}\;\text{an}\;\text{ion}\;\text{at}\;\text{equilibrium}\;\left(q_{e},mg/g\right)$$

(3) $$q_{t}=\frac{\left(C_{0}-C_{t}\right)V}{m}\;\text{The}\;\text{amount}\;\text{of}\;\text{ions}\;\text{absorbed}\;\text{per}\;\text{unit}\;\text{mass}\;\text{of}\;\text{adsorbent}\;\text{at}\;\text{time}\;t\;\left(q_{t},mg/g\right)$$

Where $C_{0}$ the initial metal concentration (mg/L) is, $C_{e}$ is the final metal concentration (mg/L), misthemassoftheadsorbent,ingrams(g),Visthevolumeofthesolution,inliters(L).

### 2.4 Adsorption isotherms

After conducting experiments to determine the adsorption equilibrium of cadmium and mercury on pomegranate peel, the results were used to calculate the isotherm parameters for Langmuir, Freundlich, and Temkin models. The equilibrium data were then used to determine the model with the best fit.

#### 2.4.1 Langmuir isotherm

In fact, the Langmuir model is a widely used model to explain the monolayer adsorption of metal entities on the outer surface of an adsorbent. It is based on the principle that the monolayer sorption process is independent, i.e. there are no interactions between the adsorbed molecules. The Langmuir equation (Eq. 4) for monolayer adsorption is given by

(4) log(C/qmax)=log(qmax)−log(q)

where C represents the residual concentration of the metal, and qmax and q represent the maximum adsorbent capacity and the adsorbed amount of the metal, respectively. The Langmuir isotherm is often more useful in real-world situations and is also easier to use.

#### 2.4.2 Freundlich isotherm

The Freundlich isotherm model is another popular theory used to describe the multilayer sorption of metal species on a non-uniform surface. It takes into account the heterogeneity of the adsorbent surface and the fact that the sorption process is not independent. It is more complex than the Langmuir model. The Freundlich isotherm equation is as follows [Eq. (5)]:

(5) $$\log\;q_{e}=\log\;K_{f}+\left(1/n\right)\;\log\;C_{e}$$

Where $q_{e}$ is the amount of adsorbed metal ions (mg/g), $C_{e}$ is the concentration of metals in solution (mg/L), $K_{f}$ is the freundlich isotherm constant, and n is a measure of the amount of energy required for the sorption process. The intercept of the linear plot of $\log\left(q_{e}\right)$ versus $\log\left(C_{e}\right)$ is equal to $\log\left(K_{f}\right)$, and the slope of the line is equal to (1/n).

#### 2.4.3 Temkin isotherm

The Temkin isotherm model assumes that the sorption energy is distributed uniformly over the surface of the adsorbent particles. It also assumes that the energy released at the surface of the adsorbent particles decreases linearly, rather than logarithmically as in the Langmuir and Freundlich models. The Temkin model is expressed by Eq. (6):

(6) q=ATexp[b(RT)]

where A is the Temkin isotherm equilibrium constant, T is the absolute temperature, b is the Temkin isotherm constant, and q is the amount of adsorbate bound to the adsorbent surface. The equation shows that the sorption energy is linearly related to temperature and the amount of adsorbate varies exponentially with temperature.

## 3 Results and discussion

### 3.1 Optimizing parameters (pH and ionic strength)

**Fig. 3:**
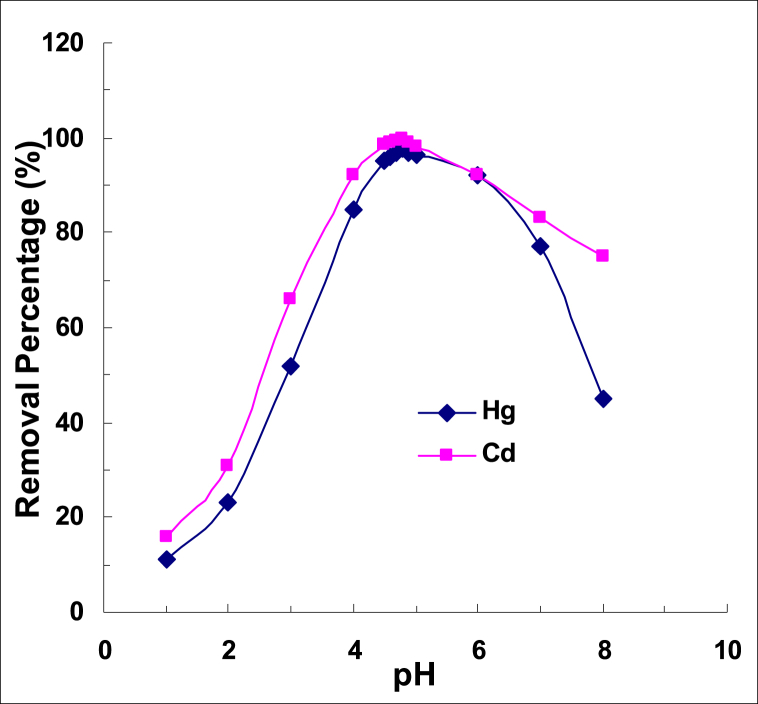
The amount of cadmium and mercury removed at different pH values.

Optimization of pH and Ionic Strength Achieved Using Continuously Operating Systems - In this section, we focus on the optimization of pH and ionic strength in the sorption of heavy metals, mercury and cadmium, using continuously operating systems. We used a variety of strategies, including discontinuous experiments, to achieve the desired results. The experimental design used in these studies involved the use of crushed pomegranate rind extract in a solution containing 25 mg/L of mercury or cadmium (II). The solution was adjusted to different pH values using hydrochloric acid or soda. We used 0.50 g of pomegranate peel extract added to 100 ml of solution. The solutions were mixed with the pomegranate peel in a 250 ml Erlenmeyer flask and shaken at 190 rpm for 220 min. After filtering the peel, the solutions were analyzed for residual mercury or cadmium using the method presented. As shown in Fig. 3, the optimum pH for this procedure is 4.8, where maximum recovery is obtained.

From the results obtained, we can see that at low pH, increasing the concentration of H^+^ ions (hydronium) causes more competition with dissolved mercury and cadmium cations, resulting in the adsorption of H^+^ by the pomegranate peel instead of mercury and cadmium. As a result, the amount of mercury and cadmium adsorbed decreases. At higher pH, with a decrease in the amount of H^+^ ions, the adsorption of mercury and cadmium ions increases, resulting in an increase in the amount of adsorption. The decrease in the amount of adsorption at pH values above 5.5 can be attributed to the presence of a higher concentration of hydroxide. As a result, based on the following reaction (Eq. (7)), cadmium (or mercury) combines with OH^−^ and precipitates strongly, to reduce the amount of cadmium that is adsorbed by the fertilized resin. The optimum pH for this process was found to be around 4.8, where maximum adsorption was achieved.

(7) $$2OH^{-}+Cd^{2+}=Cd{\left(OH\right)}_{2}$$

Hydrochloric acid and soda lye were used in all tests where it was necessary to adjust the pH of the samples to 4.8. It was observed that the ionic strength did not have a significant effect on the recovery value and a value of 0.01 was found to be optimal. For all subsequent tests and measurements, the pH of the samples was adjusted to the optimal values as previously mentioned and the ionic strength was set at 0.01. It should be noted that the removal capacity of the desired cations was not significantly reduced up to an ionic strength of 0.35.

### 3.2 Effect of contact time in discontinuous experiments

**Fig. 4:**
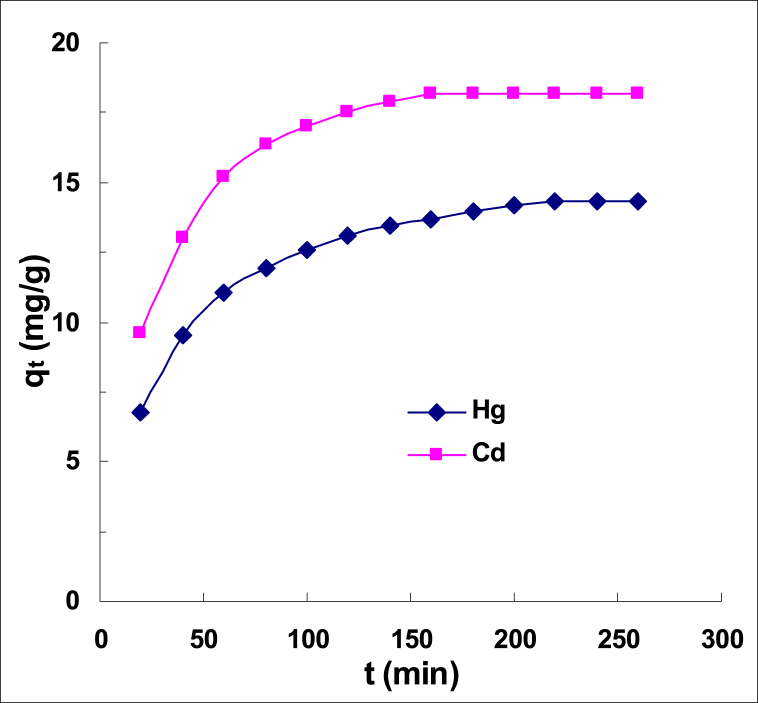
The cadmium and mercury ion adsorption capacity as a function of agitation time at optimum pH and ambient temperature.

To investigate the effect of stirring time, 100 ml solutions containing 100 mg/L cadmium or mercury and the optimal pH were first prepared. The pomegranate peel (0.50 g) was then added and the samples were shaken for varying lengths of time. After stirring, separate the adsorbent from the solution with a sieve and measure the residual solution concentration. The metal uptake was calculated and according to Fig. 4, the equilibrium time was approximately 140 min for mercury and 120 min for cadmium. As shown, extending the contact time beyond these intervals had no effect on the amount of uptake.

### 3.3 Mechanism and thermodynamics of adsorption

Adsorption isotherms are important factors in identifying suitable adsorbents by describing the relationship between the amount of solute adsorbed and its concentration in solution at constant temperature and equilibrium conditions [39,40]. Most isotherms have been used to model the absorption of solutes on an adsorbent, with the Langmuir, Freundlich, and Temkin models being the most popular. In the present study, we aimed to determine the most appropriate isotherm model to explain the absorption of mercury and cadmium on pomegranate skin. The Langmuir and Freundlich isothermal models, whose equations are given below (Eqs. (8)–(13)), were used to perform the equilibrium tests to fit the experimental data:

Langmuir model

(8) $$q_{e}=\frac{q_{\max}bC_{e}}{1+bC_{e}}$$

Freundlich model

(9) $$q_{e}=K_{F}C_{e}^{1/n}$$

Temkin model

(10) $$q_{e}=\frac{RT}{b}\left(\ln\;K_{T}C_{e}\right)$$

Their linear form is as follows in each case:

(11) $$\frac{C_{e}}{q_{e}}=\frac{C_{e}}{q_{\max}}+\frac{1}{bq_{\max}}$$

(12) $$\log\;q_{e}=\log\;K_{f}+\frac{1}{n}\log\;C_{e}$$

(13) $$q_{e}=B\;\ln\;K_{T}+B\;\ln\;C_{e}$$

Adsorption isotherms describe the relationship between the amount adsorbed and the concentration of a solute. Ce is the equilibrium concentration ($mmol.L^{-1}$), qe is the equilibrium adsorption capacity, qmax is the maximum adsorption capacity $\left(mmol.g^{-1}\right)$ , and B $\left(L.m{mol}^{-1}\right)$ is a parameter related to the adsorption energy. The Langmuir, Freundlich and Langmuir-Freundlich equations are used to describe these relationships. $K_{f}$ is the relative adsorption capacity $\left(m{mol}^{1-\left(1/n\right)}.\;l^{1/n}.g^{-1}\right)$, n is an experimental parameter proportional to the intensity of adsorption, (B=RT/b) is the constant associated with the heat of adsorption, and $K_{T}\left(L.g^{-1}\right)$ is the equilibrium binding constant corresponding to the maximum binding energy.

**Fig. 5:**
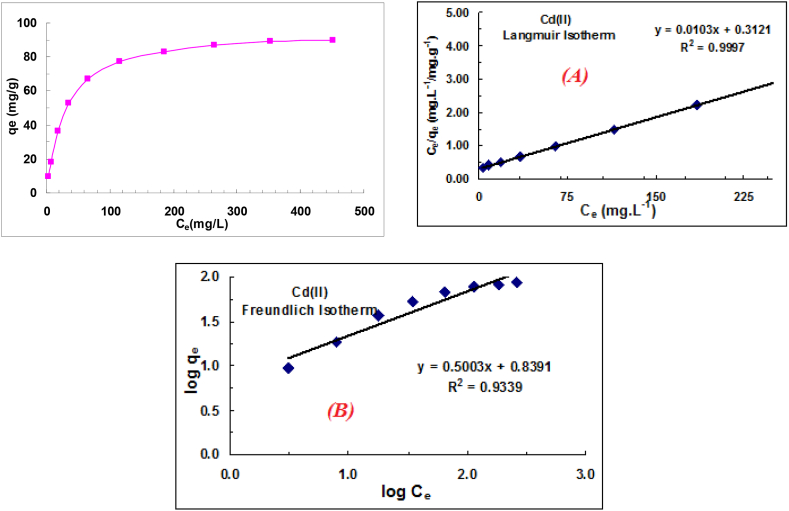
Isothermal fitting and equilibrium data for cadmium adsorption: Langmuir (A) and Freundlich isotherm (B) models.

**Fig. 6:**
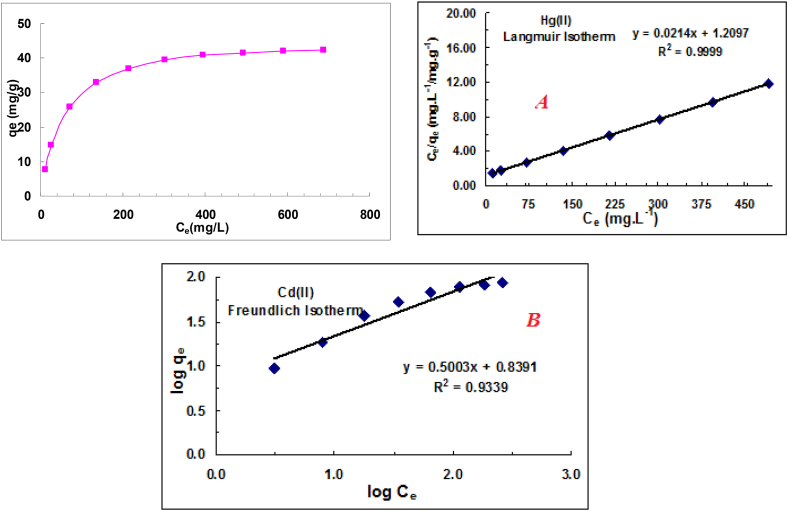
Mercury adsorption equilibrium data and isothermal fitting: Langmuir (A) and Freundlich isotherm (B) models.

Solutions of different concentrations were prepared and 0.50 g of the absorbent was added to each. Isothermal curves were then plotted based on the Freundlich and Langmuir equations, which are commonly used for isothermal studies. After complete equilibration (3 h of stirring), the samples were measured and the absorbency calculated. The difference between the initial concentration $\left(C_{0}\right)$ and the final concentration $\left(C_{e}\right)$ was used to determine the amount of cation absorbed on the adsorbent. The experimental data were then fitted with the Langmuir and Freundlich isothermal models, and the isotherm plots for cadmium and mercury were generated (Fig. 5, Fig. 6). The ambient temperature was 25 °C.

Both the Langmuir and Freundlich models were used to fit the equilibrium data obtained from the adsorption experiments. The obtained isotherm parameters were used to determine the adsorbent properties and to select the most appropriate model to describe the experimental data.

Isotherm is a term used in thermodynamics that refers to the relationship between the amount of a gas or substance absorbed by the surface of a material and the temperature at which this absorption occurs. In the context of adsorption, an isotherm describes the relationship between the concentration of a substance in a solution and the amount of the substance absorbed by a specific absorbent material at a constant temperature.

Langmuir parameters for the absorption of ions, along with those of other models, have been calculated and are presented in Table 2, Table 3. These parameters provide insight into the properties of the adsorbent and the interactions between the ions and the adsorbent particles. The Langmuir equation was the most appropriate model to describe the adsorption of the ions on the pomegranate peel.

**Table 2:** Calculated isotherm parameters for cadmium ion adsorption.

Freundlich isotherm parameters			Langmuir isotherm parameters		
$K_{f}$, (mmol^1−(1/n)^.L^1/n^.g^−1^)	1/n	R^2^	b, L/mg	$q_{m}$, mg/g Calculated/experimental	R^2^
6.90	0.5003	0.9339	0.033	97.09/89.59	0.9997

**Table 3:** Calculated isotherm parameters for mercury ion adsorption.

Freundlich isotherm parameters			Langmuir isotherm parameters		
$K_{f}$, (mmol^1−(1/n)^.L^1/n^.g^−1^)	1/n	R^2^	b, L/mg	$q_{m},mg/g$ Calculated/experimental	R^2^
2.73	0.5205	0.9374	0.018	46.73/42.25	0.9999

Isotherms are essential tools for gaining insight into the thermodynamics of the adsorption process. They provide information about the interaction between the contaminant and the adsorbent materials and the structure of the adsorption layer. The shape and change of the isotherm can provide valuable information about the mechanism and strength of the adsorbent-adsorbate interactions. In this study, three isothermal models, Langmuir, Freundlich and Temkin, were used to fit the adsorption equilibrium data. The calculated parameters in Table 2, Table 3 showed satisfactory fits for both the Freundlich and Langmuir models, with the Langmuir model showing better fit capability. This suggests that the cadmium adsorption on the pomegranate peel-derived adsorbents was a monolayer process, involving homogeneous active sites, and a binding process influenced by various chemical and physical interactions. Further analysis based on the Langmuir model revealed that the theoretical adsorption capacity of cadmium ions (89.59 mg/g) was 112 % higher than that of mercury ions (42.25 mg/g).

## 4 Conclusions

This experiment and modeling was aimed at evaluating the performance of pomegranate peel as a novel adsorbent for the removal of cadmium and mercury ions from wastewater. The results showed that:

1. Pomegranate peel emerges as a promising substrate for the efficient removal of both cadmium and mercury ions from water, underscoring its viability for the effective treatment of toxic pollutants.
2. Through our investigation, we have determined the optimal pH value for maximum toxin removal efficiency to be 4.8
3. Among the models studied, the Langmuir model emerges as the most suitable to accurately describe the monolayer behavior of the adsorption process in the systems studied.
4. Our results suggest a significant potential for the industrial use of pomegranate peel as an economical and efficient adsorbent material for the removal of toxic ions from wastewater.
5. In conclusion, these results provide a valuable data set for the development of sustainable and cost-effective methods for the remediation of heavy metal contaminated water.

## Additional information

No additional information is available for this paper.

## Data availability statement

Data will be made available on request.

## CRediT authorship contribution statement

**Javad Zareei:** Writing – review & editing, Writing – original draft, Visualization, Validation, Supervision, Software, Resources, Project administration, Methodology, Investigation, Funding acquisition, Formal analysis, Data curation, Conceptualization. **Nizomiddin Juraev:** Writing – review & editing, Writing – original draft, Software, Resources, Investigation. **Sabir Tagelsir Hassan Widatalla:** Writing – review & editing, Writing – original draft, Software, Resources, Investigation. **M. Kerwad:** Writing – review & editing, Writing – original draft, Software, Resources, Investigation. **Bokov Dmitry Olegovich:** Writing – review & editing, Writing – original draft, Visualization, Validation, Software, Resources, Investigation, Conceptualization. **Khalid A. Alkhuzai:** Writing – review & editing, Writing – original draft, Software, Resources, Investigation. **Carlos Rodriguez-Benites:** Writing – review & editing, Writing – original draft. **Merwa Alhadrawi:** Writing – review & editing, Writing – original draft. **Salah Hassan Zain Al-Abdeen:** Writing – review & editing, Writing – original draft, Visualization, Validation.

## Declaration of competing interest

The authors declare that they have no known competing financial interests or personal relationships that could have appeared to influence the work reported in this paper.
